# The Delicate Balance: Aptitude of Physicians with Psychiatric diseases

**DOI:** 10.1192/j.eurpsy.2024.1721

**Published:** 2024-08-27

**Authors:** N. Khaterchi, G. Bahri, I. Youssef, M. Mersni, H. Ben Said, D. Brahim, N. Mechergui, M. Methni, C. Ben Said, N. Bram, N. Ladhari

**Affiliations:** ^1^occupationnal medecine; ^2^ Charles Nicolle Hospital; ^3^2Forensic Psychiatry departement, Razi Hospital, La Manouba, Tunis, Tunisia

## Abstract

**Introduction:**

The delicate balance between the need to ensure quality patient care and the reality of physicians dealing with psychiatric diseases poses a major challenge within the medical field. This issue raises fundamental ethical, legal, and medical questions, highlighting the complexity of decision-making regarding professional aptitude for practitioners affected by mental disorders.

**Objectives:**

To examine the impact of psychiatric diseases on the medical aptitude of physicians.

**Methods:**

This was a retrospective descriptive study that focused on physicians with psychiatric diseases referred to the occupational pathology clinic at Charles Nicolle Hospital in Tunis for medical evaluations of their work aptitude between January 1, 2021, and September 15, 2023.

**Results:**

During the study period, we collected data from 20 patients. The mean age was 38 ± 11 years, with a sex-ratio (F/M)of 4.5. Five examined physicians had family histories of psychiatric disorders. Medical specialties were the most represented (N=17), including three general practitioners, two family medicine practitioners, and two anesthesiologists. The study population included 10 residents, eight hospital assistants, and two medical interns. The most common psychiatric diagnosis was depression (N=7), followed by bipolar disorder (N=5). The medical treatment prescribed was combinations of antidepressants and anxiolytics in seven cases, antipsychotics in five cases, and antidepressants in two cases. Medication adherence was noted in 10 physicians. Fourteen physicians had taken long-term sick leave, with an average duration of 203 days. Five physicians were declared fit to continue their regular professional activities, seven physicians were declared fit with restrictions on night work, and one physician was declared fit with workplace accommodations.

**Conclusions:**

This study highlights the challenges surrounding the medical aptitude of physicians with psychiatric diseases. However, it is imperative to promote mental health awareness and to implementsupport measures to ensure both compassion for physicians and patient safety.

**Disclosure of Interest:**

None Declared

